# Functional Mechanisms of Mitochondrial Respiratory Chain Supercomplex Assembly Factors and Their Involvement in Muscle Quality

**DOI:** 10.3390/ijms21093182

**Published:** 2020-04-30

**Authors:** Kotaro Azuma, Kazuhiro Ikeda, Satoshi Inoue

**Affiliations:** 1 Department of Systems Aging Science and Medicine, Tokyo Metropolitan Institute of Gerontology, Itabashi-ku, Tokyo 173-0015, Japan; azumak@tmig.or.jp; 2 Division of Gene Regulation and Signal Transduction, Research Center for Genomic Medicine, Saitama Medical University, Hidaka, Saitama 350-1241, Japan; ikeda@saitama-med.ac.jp

**Keywords:** mitochondria, respiratory chain supercomplex, skeletal muscle, sarcopenia, adipose tissue

## Abstract

Impairment of skeletal muscle function causes disabilities in elderly people. Therefore, in an aged society, prevention and treatment of sarcopenia are important for expanding healthy life expectancy. In addition to aging, adipose tissue disfunction and inflammation also contribute to the pathogenesis of sarcopenia by causing the combined state called ‘sarcopenic obesity’. Muscle quality as well as muscle mass contributes to muscle strength and physical performance. Mitochondria in the skeletal muscles affect muscle quality by regulating the production of energy and reactive oxygen species. A certain portion of the mitochondrial respiratory chain complexes form a higher-order structure called a “supercomplex”, which plays important roles in efficient energy production, stabilization of respiratory chain complex I, and prevention of reactive oxygen species (ROS) generation. Several molecules including phospholipids, proteins, and certain chemicals are known to promote or stabilize mitochondrial respiratory chain supercomplex assembly directly or indirectly. In this article, we review the distinct mechanisms underlying the promotion or stabilization of mitochondrial respiratory chain supercomplex assembly by supercomplex assembly factors. Further, we introduce regulatory pathways of mitochondrial respiratory chain supercomplex assembly and discuss the roles of supercomplex assembly factors and regulatory pathways in skeletal muscles and adipose tissues, believing that this will lead to discovery of potential targets for prevention and treatment of muscle disorders such as sarcopenia.

## 1. Introduction

In eukaryotic cells, mitochondria play important roles in efficient production of ATP through a process called oxidative phosphorylation (OXPHOS). OXPHOS is carried out by five molecular complexes embedded in the inner mitochondrial membrane, namely, complex I (NADH ubiquinone oxidoreductase/NADH dehydrogenase), complex II (succinate ubiquinone oxidoreductase/succinate dehydrogenase), complex III (ubiquinol cytochrome *c* oxidoreductase/cytochrome *bc*_1_ complex), complex IV (cytochrome *c* oxidase), and complex V (ATP synthase). Complexes I–IV constitute the respiratory electron transport chain and are responsible for transferring electrons from NADH or FADH2 to molecular oxygen. In the process, protons translocate across the inner mitochondrial membrane from the mitochondrial matrix to the intermembrane space. Thus, a proton gradient is established across the inner membrane, which is essential for ATP generation by complex V.

It has been recently reported that a certain portion of the mitochondrial respiratory chain complexes form a higher order structure called a “supercomplex” [1]. Cryo-electron microscopic studies and blue native PAGE analysis using digitonin-extracted mitochondria reveal that complexes I, III, and IV are involved in the formation of supercomplexes. There are several combinations of supercomplexes including I/III_2_, I/III_2_/IV_n_ (*n* = 1–4), and III_2_/IV_n_ (*n* = 1 or 2) [2]. The supercomplex type I/III_2_/IV_n_ is named ‘respirasome’ [1]. The biological significance of the mitochondrial respiratory chain supercomplexes is efficient energy production [3,4,5], stabilization of complex I [6], and prevention of reactive oxygen species (ROS) generation [7,8].

In this review, we discuss the biological significance of mitochondrial respiratory chain supercomplex formation in muscular tissues and in adipose tissues. As related diseases, obesity and sarcopenia are mentioned. During exercise, skeletal muscles consume a large proportion of energy produced by the mitochondria. Research on animal models and clinical findings imply that formation of mitochondrial respiratory chain supercomplexes is favorable for exercise. Moreover, several molecules affecting mitochondrial respiratory chain supercomplex assembly and stability were identified, leading to deeper understanding of supercomplex formation and its regulation. The roles of mitochondrial respiratory chain supercomplex formation have not been studied in adipose tissues in comparison with muscular tissues, but functions of some of the supercomplex assembly factors suggest its importance in adipose tissues as well. Indeed, adipose tissue disfunction and inflammation cause the combined state called ‘sarcopenic obesity’ with elevated risks of disability [9].

## 2. Supercomplex Assembly Factors and Molecules Affecting Supercomplex Formation

Mitochondrial respiratory chain supercomplex assembly has been studied in yeasts, plants, and mammals, and it has been found that the respiratory complexes of each are different. For example, complex I is generally absent from the mitochondria of yeasts (with some exception [10]). Some supercomplex assembly factors are evolutionary conserved in different species whereas some are not. In this review, we focus on mitochondrial respiratory chain supercomplex formation in mammalian cells, especially from mice and humans, as they possess the muscular tissues and output of its function can be evaluated as several parameters of exercise.

**Figure 1:**
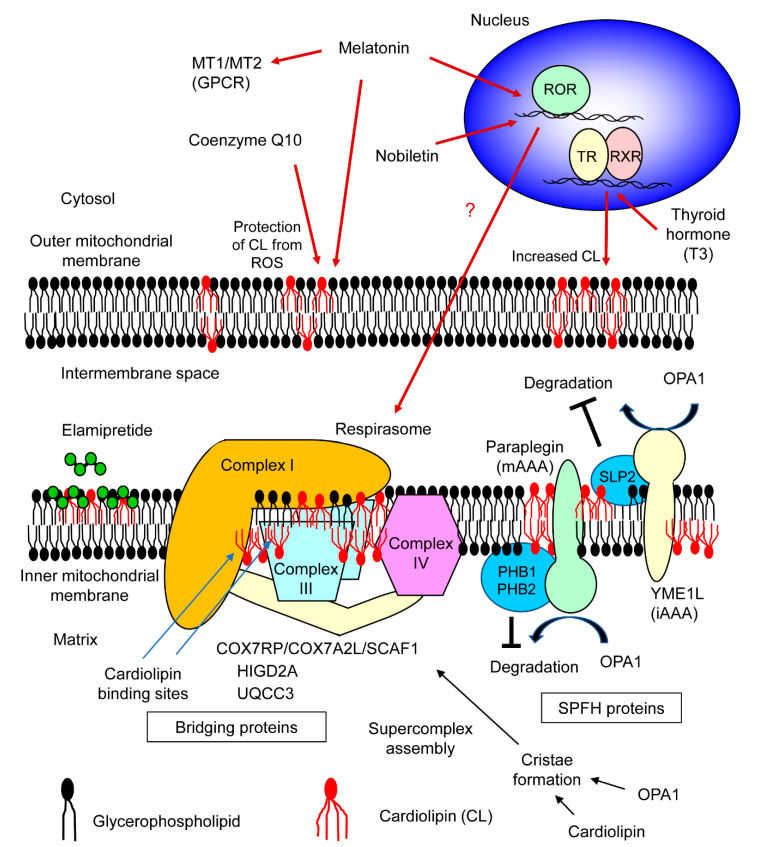
Molecules involved in mitochondrial respiratory chain supercomplex assembly. The respiratory chain supercomplex assembly factors and molecules affecting supercomplex formation which are reported to function in mitochondria of vertebrates are shown. Cardiolipin (CL), a phospholipid with four acyl chains, directly binds respiratory chain complexes on the cardiolipin binding sites in each complex. Cardiolipin may indirectly support mitochondrial respiratory chain supercomplex formation by maintaining cristae structure. COX7RP (cytochrome c oxidase subunit 7a-related polypeptide; also known as COX7A2L/SCAF1), HIGD2A (HIG1 hypoxia inducible domain family, member 2A), and UQCC3 (ubiquinol-cytochrome c reductase complex assembly factor 3) function as bridging proteins by direct binding with each respiratory chain complex. PHB1 (prohibitin 1), PHB2 (prohibitin 2), and SLP2 (stomatin-like protein 2) are SPFH (stomatin, prohibitin, flotillin, and Hflk/C) proteins which inhibit activity of proteases, mAAA (a member of AAA (ATPase associated with diverse cellular activities) proteins with enzymatic sites in the matrix of mitochondria) and iAAA (a member of AAA proteins with enzymatic sites in the intermembrane space of mitochondria), causing degradation of OPA1 (optic atrophy 1) responsible for the maintenance of cristae structure. Five bioactive small molecules are also shown. Coenzyme Q10 and melatonin are antioxidants that protect cardiolipin from reactive oxygen species (ROS). Functions of melatonin are also mediated by its G-protein coupled receptors (MT1 and MT2) or by nuclear receptor ROR (retinoid acid receptor-related orphan receptor). Nobiletin also activates ROR. T3 type of thyroid hormone binds to thyroid hormone receptor (TR). TR forms heterodimer with retinoid X receptor (RXR). T3 increases the amount of cardiolipin. Elamipretide is a synthetic tetrapeptide which modulates electrostatics of the mitochondrial inner membrane.

Cardiolipin is an acidic phospholipid having a unique structure comprised of two phosphate groups and four acyl chains. Cardiolipins are exclusively localized in mitochondria. In mitochondria, they play a role in generating structures such as cristae [11] and contact sites [12] where the inner membrane and outer membrane of mitochondria are fused. Though, it was known that cardiolipin stabilizes complex III- and complex IV-containing supercomplexes in yeasts [13], the importance of cardiolipin in human mitochondrial respiratory chain supercomplex assembly was shown by the analysis of primary lymphoblasts derived from Barth syndrome patients. Barth syndrome is an X-linked hereditary disease caused by a mutation in the *TAZ* gene which encodes Taffazin [14], an acyltransferase responsible for cardiolipin remodeling [15]. In normal individuals, cardiolipins having four linoleic acids (called tetralinoleoylcardiolipin) are present; whereas Barth syndrome patients show abnormal accumulation of the precursor form of tetralinoleoylcardiolipin (called monolyso-cardiolipin) [16,17,18]. Blue native PAGE analysis revealed that in the lymphoblasts of Barth syndrome patients, where insufficient mature cardiolipin existed, assembly of I/III_2_/IV-type supercomplex (respirasome) decreased [14], indicating that abundant mature cardiolipin is essential for mitochondrial respiratory chain supercomplex formation. Later, it was shown that cardiolipin has high affinity towards specific ‘cardiolipin binding sites’ enriched in positive amino acids on the membrane-exposed surface of respiratory complexes [19]. Thus, cardiolipin functions as a bridge between cardiolipin binding sites of different respiratory complexes (Figure 1). Comparison of the binding strength of cardiolipin with that of other lipids revealed that the binding strength of cardiolipin was significantly higher [19].

Besides phospholipids, several additional protein factors involved in mitochondrial respiratory chain supercomplex formation and stabilization have been discovered. A mitochondrial protein, HIGD2A (HIG1 hypoxia inducible domain family, member 2A), was shown to have a supercomplex stabilizing effect in murine myoblastic cells, C2C12 [20]. This study primarily confirmed that respiratory supercomplex factor 1 (Rcf1) protein interacted with complexes III and IV of mitochondrial electron transport chain independently and stabilized the III/IV supercomplex in yeast cells. Moreover, this study investigated the mammalian homologs of yeast Rcf1. In C2C12 cells, knockdown of *Higd2a*, a homolog of Rcf1, impaired the formation of mitochondrial respiratory chain supercomplexes including respirasome (Figure 1). It is interesting that HIGD2A affects the formation of complex I-containing supercomplexes because its evolutionary ancestor Rcf1 is involved only in the formation of complex III- and complex IV-containing supercomplexes in yeast mitochondria lacking complex I. Another study reported that HIGD2A is induced by hypoxia not only in murine C2C12 cells, but also in human embryonic kidney cells [21]. However, the mitochondrial respiratory chain supercomplex assembly-promoting effect of HIGD2A in human cells was not evident in this study.

COX7RP (cytochrome *c* oxidase subunit 7a-related polypeptide; also known as COX7A2L/SCAF1), which was originally identified as a protein induced by estrogen in breast cancer cells [22], was found to function as a mitochondrial respiratory chain supercomplex assembly-promoting factor in skeletal muscles derived from *Cox7rp*-knockout mice [3]. Blue native PAGE analysis revealed that formation of supercomplex I/III_2_/IV_n_ was impaired in the skeletal muscles of *Cox7rp*-knockout mice. Mitochondrial respiratory chain supercomplex assembly-promoting effect was also observed in human embryonic kidney HEK293T cells [3], human breast cancer MCF-7 cells and human endometrial cancer Ishikawa cells [23], indicating that COX7RP may function as a supercomplex assembly-promoting factor in human cells as well. The oxygen consumption rate of murine embryonic fibroblasts (MEFs) derived from *Cox7rp*-knockout mice was decreased in a medium containing pyruvate and malate (for which electrons are carried by NADH) and in a medium containing succinate (for which electrons are carried by FAD), indicating that mitochondrial respiratory chain supercomplex formation enhanced respiratory chain activity. Simultaneously, the importance of COX7RP was independently reported by other researchers by screening of proteins present in the mitochondrial respiratory chain supercomplexes [4]. Less mitochondrial respiratory chain supercomplex formation was observed in several tissues (such as liver) of C57BL/6J and Balb/c mice, which possessed a short form COX7RP variant, compared to strains (CBA, 129, NZB, and CD1) having a long form COX7RP variant [4,24]. An additive effect of pyruvate and malate treatment and succinate treatment on respiration was shown in liver mitochondria having the long form COX7RP [4]. However, the additive effect was weak in mitochondria having the short form of COX7RP, confirming the role of COX7RP in respiratory chain activation. In addition, little difference was observed in respiratory chain supercomplex formation of heart mitochondria between mice strains, suggesting tissue-specific mechanism of supercomplex assembly [25,26]. Later, insights on the mechanism of supercomplex assembly-promoting effect by COX7RP have been provided by other studies. Analysis of the interaction between COX7RP and respiratory chain complexes by three-dimensional models indicates that COX7RP can bind both complexes I and III with salt bridges [25]. Blue native PAGE analysis of COX7RP variants reveals that His73 of COX7RP is critical for its interaction with complex IV. This study also revealed that the region near N-terminus of COX7RP is responsible for the formation of supercomplexes I/III_2_ and III_2_/IV. All these reports indicate that COX7RP functions as a scaffold for mitochondrial respiratory chain supercomplex assembly by physical interaction with its pleural regions (Figure 1). Indeed, Pro71 and Ile72 of COX7RP locating just next to His73 were absent from the short form of COX7RP variant [25], which may explain the different function of the two variants. Despite many lines of evidence revealing the function of COX7RP, there are a few reports denying the involvement of complex I in the supercomplex promoted by COX7RP [26,27]. From the phylogenetic point of view, the *COX7RP* gene is evolutionarily conserved only in vertebrates [28] in contrast to *HIGD2A* which is conserved in all eukaryotes [17].

UQCC3 (ubiquinol-cytochrome *c* reductase complex assembly factor 3; also known as C11orf83) is a mitochondrial protein which is highly conserved in metazoans. The function of UQCC3 in humans was studied using HeLa cells derived from human cervical cancer. Blue native PAGE analysis showed that knockdown of *Uqcc3* leads to a decrease in supercomplexes I/III_2_/IV (respirasome) and III_2_/IV, indicating that UQCC3 has supercomplex assembly-promoting effect in human cells [29]. Further study revealed that UQCC3 has affinity towards III_2_-type supercomplex and cardiolipin, suggesting that UQCC3 has a role in supercomplex formation and stabilization (Figure 1).

SLP2 (stomatin-like protein 2) is a mitochondrial protein conserved in eukaryotes, which was reported to have a respiratory chain supercomplex assembly-promoting effect in MEFs. Blue native PAGE analysis revealed that formation of I/III_2_/IV_n_-type supercomplex (respirasome) was decreased in MEFs lacking SLP2, without affecting the formation of individual complexes I, III, and IV [30]. Since SLP2 forms cardiolipin-enriched microdomains in the inner mitochondrial membrane [31], the respiratory chain supercomplex assembly-promoting effect of SLP2 is suggested to be dependent on cardiolipin. Another possible mechanism is the association of SLP2 with YME1L, an iAAA protease (a member of AAA (ATPase associated with diverse cellular activities) proteins with enzymatic sites in the intermembrane space of mitochondria) functioning in the intermembrane space of mitochondria [32] (Figure 1). YME1L cleaves OPA1 (optic atrophy 1) [33] which plays a role in maintaining the shape of mitochondrial cristae. It has been reported that acute ablation of *Opa1* in murine fibroblastic cells impaired mitochondrial respiratory chain supercomplex assembly, while fibroblastic cells derived from *Opa1* transgenic mice displayed enhanced supercomplex formation [34]. This indicates that the shape of mitochondrial cristae is an important determinant of respiratory chain supercomplex stability, and thus, OPA1 protein indirectly regulates supercomplex assembly.

Two evolutionarily conserved proteins in yeast, PHB1 (prohibitin 1) and PHB2 (prohibitin 2), are added to the list of mitochondrial respiratory chain supercomplex assembly-promoting factors. In HeLa cells, knockdown of either *Phb1* or *Phb2* reduced the formation of supercomplexes I/III_2_/IVn (respirasome) and III_2_/IV, without affecting the formation of individual complexes I, III, and IV [35]. Though the underlying mechanism was not discussed in the same study, a mechanism similar to that of SLP2 has been speculated as prohibitins and SLP2 share a common domain called SPFH (stomatin, prohibitin, flotillin, and Hflk/C) domain, and both are classified as members of the SPFH protein family. Members of the SPFH protein family share the characteristics of scaffold proteins which locally specify the protein-lipid composition in the membranes. In contrast to SLP2 which forms a complex with iAAA protease, PHB1 and PHB2 interact with mAAA protease (a member of AAA proteins with enzymatic sites in the matrix of mitochondria) [36] (Figure 1). Here, OPA1 functions as a substrate for paraplegin (an mAAA protease) [37], suggesting the same speculation that the functions of PHB1 and PHB2 are similar to that of SLP2.

Some bioactive small molecules including intrinsic hormones, a flavonoid in plants, and an artificial compound are known to promote mitochondrial respiratory chain supercomplex formation. Coenzyme Q10, a fat-soluble small molecule naturally synthesized in cells, is one of them. Coenzyme Q10 is a cofactor for mitochondrial electron transport chain. In a mouse model of burn injury, which was previously shown to induce mitochondrial dysfunction, there was a decrease in supercomplexes I/III_2_/IV_n_ (respirasome) and III_2_/IV_n_ in mitochondria isolated from rectus abdominis muscle which was not directly affected by burn as compared to mitochondria isolated from mice treated with sham burn. Administration of coenzyme Q10 restored the formation of mitochondrial respiratory chain supercomplexes [38]. Burn induced loss of cristae structure in skeletal muscle mitochondria, which was restored by coenzyme Q10 administration. This observation may be related to the mechanism of coenzyme Q10 in mitochondrial respiratory chain supercomplex formation, though upregulation of OPA1 by coenzyme Q10 was not detected in this study [38].

Melatonin is a small molecule synthesized form tryptophan. In vertebrates, it is mainly produced in the pineal gland and functions as a hormone with pleiotropic effect including regulating circadian rhythm, sleep, and immune function. However, melatonin is an evolutionary old molecule and is synthesized by some kinds of bacteria. Melatonin has multiple modes of function. Melatonin itself functions as direct radical scavenger of various reactive species including ROS [39], which may explain its effect on mitochondrial respiratory chain supercomplex explained later (Figure 1). Alternatively, functions of melatonin can be mediated by its specific G protein-coupled receptors (GPCRs) called MT1 and MT2. Melatonin could also activate nuclear receptor ROR (retinoid acid receptor-related orphan receptor) α [40]. In the sepsis model of mice, administration of melatonin prevented sepsis-dependent decline of mitochondrial respiratory function [41]. This effect was accompanied by increase of supercomplexes formation containing complex III, but not ones containing complex I.

Another hormone, thyroid hormone, is shown to promote the mitochondrial respiratory chain supercomplex. In the experiments using rats, hypothyroidism induced by propylthiouracil and iopanoic acid caused reduced formation of respiratory chain supercomplex in the mitochondria in the liver, and administration of T3 (3,5,3’-triiodo-L-tyronine) type of thyroid hormone rescued the formation of respiratory chain supercomplex [42]. Functions of thyroid hormone is mediated by the thyroid hormone receptor (TR), which usually forms a heterodimer with retinoid X receptor (RXR). Both TR and RXR belong to the nuclear receptor superfamily. As a consequence of stimulation of thyroid hormone, increase of cardiolipin levels are observed in mouse skeletal muscle [43]. This may be a mechanistic link between thyroid hormone and mitochondrial respiratory chain supercomplex (Figure 1).

Nobiletin is a naturally existing flavonoid contained in citrus peels. Nobiletin is known to activate nuclear receptor RORα and γ [44]. In the skeletal muscle of aged mice, the mitochondrial respiratory chain supercomplex I_n_/III_n_, which contains multiple complex I and no complex IV, is decreased by high-fat diet. Nobiletin treatment restored I_n_/III_n_-type supercomplex; although, this result was circadian rhythm-dependent, that is, observed only when the muscle sample was obtained during the period when the mice were inactive [45]. The effect of nobiletin implies that mitochondrial respiratory chain supercomplex formation is affected by ROR signaling. It is interesting that nobiletin and melatonin share the relationship with circadian rhythm, and both can function thorough RORα (Figure 1).

Elamipretide (D-Arg-2’6’-dimety Tyr-Lys-Phe-NH2) is a synthetic tetrapeptide with alternating cationic and aromatic residues. It is cell-permeable and accumulated at the mitochondrial inner membrane. When human heart tissues derived from explanted hearts of heart failure patients were treated with elamipretide, mitochondrial respiratory chain supercomplex containing complex IV was increased without affecting cardiolipin content [46]. Modulating electrostatics of the mitochondrial membrane is suggested as a mechanistic explanation of elamipretide by using an artificial membrane model [47] (Figure 1).

## 3. Mitochondrial Respiratory Chain Supercomplex Assembly and Sarcopenia

The word sarcopenia, which means ‘muscle loss’ in Greek, was invented by Dr. Rosenberg in 1988 to indicate age-associated muscle weakness [48]. In 2010, the operational definition of sarcopenia was proposed to identify people with pathological muscle loss [49]. In 2016, sarcopenia was added to the list of International Classification of Diseases (ICD)-10, indicating that sarcopenia became known as a disease. In this decade, several diagnostic criteria were proposed and several studies on sarcopenia were undertaken worldwide. Among those criteria, the latest one emphasized muscle strength and physical performance rather than muscle mass [50], based on the notion that attention must be paid to muscle quality as well as muscle mass. Mitochondria in the skeletal muscle are regarded as important determinants of muscle quality because they produce energy required for muscle contraction. It has been reported that aging decreases mitochondrial respiratory chain supercomplex formation in rat hearts [51]. In humans, age-associated decline in *OPA1* expression was observed in sedentary seniors, with concomitant muscle weakness [52]. Considering the role of OPA1 in mitochondrial respiratory chain supercomplex formation [34], it can be inferred that there is an association between supercomplex formation and sarcopenia; therefore, supercomplex assembly factors and molecules affecting supercomplex formation are potential targets for sarcopenia treatment. In addition, we would like to mention the roles of supercomplex assembly factors in adipose tissues considering the disfunction and inflammation of adipose tissues can be causes of sarcopenia [9].

## 4. Roles of Supercomplex Assembly Factors and Molecules Affecting Supercomplex Formation in Skeletal Muscle and Adipose Tissues

The symptoms of Barth syndrome provide certain clues regarding the function of cardiolipin in skeletal muscles. The main clinical features of Barth syndrome are dilated cardiomyopathy, myopathy, and neutropenia [53], indicating that cardiolipin abnormality affects skeletal muscles. Proximal myopathy and exercise intolerance were also reported in these patients. In addition to respiratory chain supercomplex stabilization, cardiolipin has other mitochondrial functions such as fusion of mitochondria [54] and regulation of apoptotic signals [55]; therefore, it cannot be concluded that all the phenotypes of Barth syndrome result from defective supercomplex formation. The functions of cardiolipin in adipose tissues were demonstrated by generating mice with adipose tissue-specific knockout of cardiolipin synthase 1 (*Crls1*) gene. Adipose tissue-specific *Crls1*-knockout mice exhibited decreased cardiolipin content in brown adipose tissue (BAT) and impaired resistance to cold exposure [56]. Interestingly, cardiolipin was prominently induced by cold exposure in BAT accompanied by induction of CRLS1. Adipose tissue-specific *Crls1*-knockout mice showed altered glucose metabolism as well. Insulin resistance was observed in glucose tolerance test. This phenotype was also present in BAT-specific *Crls1*-knockout mice [56], suggesting importance of cardiolipin in BAT on systemic glucose metabolism.

*Higd2a* expression analysis in several mouse tissues revealed that *Higd2a* was most abundantly expressed in skeletal muscle followed by heart muscle [21]. This implies that HIGD2A has important roles in the skeletal muscle. It was also shown that *Higd2a* expression was induced by hypoxia and low glucose conditions in C2C12 myoblastic cells [21]. Thus, HIGD2A mediates the regulation of mitochondrial respiratory chain supercomplex assembly in response to metabolic change during exercise.

In the forced treadmill test, *COX7RP*-transgenic mice exhibited significantly enhanced running capacity, while *Cox7rp*-knockout mice showed significantly lower running capacity [3]. Although this effect could not be entirely attributed to the degree of mitochondrial respiratory chain supercomplex assembly, the treadmill test of these animal models suggests that the supercomplex assembly factor COX7RP could affect muscle quality. *Cox7rp*-knockout mice also showed altered lipid metabolism in BAT [3] and glucose metabolism in liver [57]. The BAT in *Cox7rp*-knockout mice looked pale and contains larger lipid droplet. The resistance to cold exposure was impaired in *Cox7rp*-knockout mice, indicating decreased thermogenesis. In the liver of *Cox7rp*-knockout mice, gluconeogenesis was impaired, which may result in insufficient glucose supply for skeletal muscle.

For UQCC3, there is a case report on a patient from consanguineous parents with homozygous missense mutation of *Uqcc3*. The patient had persistent muscular weakness and increased fatigability [58], suggesting the importance of UQCC3 in skeletal muscle.

Systemic knockout of *Slp2* was reported to be embryonic lethal in mice [59]. To our best knowledge, there is no literature describing the function of SLP2 in skeletal muscles. As SLP2 is expressed in skeletal muscles according to the human protein atlas [60], it is speculated that SLP2 has some roles in exercise.

After prohibitins were shown to promote mitochondrial respiratory chain supercomplex formation, PHB2 was reported to play an important role in mitophagy [61]. Mitophagy is a process in which damaged mitochondria are removed by autophagy; this prevents release of reactive oxygen species from defective mitochondria. The hereditary Parkinson’s disease-causing proteins PINK1 (PTEN-induced putative kinase 1) and Parkin play a role in regulating mitophagy, indicating the physiological importance of mitophagy in preventing neurodegenerative disorders. Age-dependent decrease in PINK1 and Parkin expression in rat skeletal muscle and human skeletal muscle, respectively [62,63], suggests that inefficient mitophagy could be one of the causes of sarcopenia. The discovery that PHB2 is required for Parkin-mediated mitophagy suggests that PHB2 is involved in the maintenance of muscle quality by a different mechanism. Interestingly, PHB2 lacking the domain responsible for interaction with autophagosome-associated protein LC3 caused impairment of mitophagy, while the same mutation did not affect OPA1 stabilization [61]. This suggests that inhibition of mAAA and mitophagy are the distinct functions of PHB2. As far as we know, no literature describes the function of prohibitins in skeletal muscle, except one report on describing the involvement of prohibitins in myogenesis. Nevertheless, accumulating evidence on prohibitins’ functions in other tissues suggests that prohibitins have certain functions in muscular mitochondria. On the other hand, prohibitins play important roles in adipose tissues. Induction of both *Phb1* and *Phb2* was observed during adipocytic differentiation of 3T3-L1 murine preadipocytes, and suppression of PHB1 or PHB2 impaired adipogenesis [64]. The adipocyte-specific *Phb1*-transgenic mice displayed obesity and insulin resistance [65]. Interestingly, the phenotype was only observed in male transgenic mice.

The relationship between coenzyme Q10 status and physical performance in humans was revealed by an epidemiological study [66]. In this study coenzyme Q10 status was evaluated as the ratio of total blood coenzyme Q10 (both oxidized and reduced forms) to serum cholesterol concentration, as both cholesterol and coenzyme Q10 are transported in lipoproteins [67], and cholesterol and coenzyme Q10 share a common synthetic pathway. A significant positive association between hand grip and coenzyme Q10 status (coenzyme Q10:cholesterol ratio) was observed [66], suggesting a possible beneficial effect of coenzyme Q10 on upper limb muscle strength. In another study, the effect of ubiquinol (reduced form of coenzyme Q10) supplementation was evaluated in young and healthy elite trained athletes in a double-blind manner [68]. After 6 weeks of ubiquinol supplementation, the athletes displayed significantly enhanced peak power production in comparison to the placebo group. This result suggested the beneficial effect of coenzyme Q10 in skeletal muscles. Part of the mechanism could be the enhancement of mitochondrial respiratory chain supercomplex formation by coenzyme Q10 [38]. From the viewpoint of adipose tissues, coenzyme Q10 has a suppressive effect for adipogenesis. Coenzyme Q10 is shown to suppress adipogenic differentiation of 3T3-L1 cells [69]. In an animal study using KKAy diabetic mice, supplementation of coenzyme Q10 reduced weight gain without affecting food intake [70]. However, in the clinical studies, coenzyme Q10 supplementation seems to have little effect on obesity. One old intervention study with favorable results exists, but the result has never been confirmed by larger studies [71].

Some epidemiologic studies suggest a beneficial effect of melatonin for sarcopenia. The urine concentration of 6-sulfatoxymelatonin, the main melatonin metabolite, was inversely associated with the muscle mass in Korean postmenopausal women [72]. In Japanese elderly population, the urine concentration of 6-sulfatoxymelatonin was significantly associated with grip strength and quadriceps strength [73]. There are many studies concerning the effects of melatonin in muscle including protective effect of muscle stem cells and suppressive effect on inflammatory response [74]. The promotive effect on mitochondrial respiratory chain supercomplex formation could be added to those mechanisms. In terms of the relationship of melatonin and obesity, a nonsynonymous single nucleotide polymorphism (resulting in amino acid variation) in the *MTNR1B* gene coding one of the melatonin receptors, MT1, was reported to be associated with increased BMI [75]. In some clinical trials, modest reduction in body weight was observed, suggesting an overall modest effect of melatonin on obesity [76].

The relationship of thyroid hormone and sarcopenia is suggested by a clinical study conducted in China. Blood concentration of free triiodothyronine (fT3) was positively associated with appendicular skeletal muscle mass, handgrip strength, and physical function assessed by the Short Physical Performance Battery (SPPB) [77]. In this study, blood concentrations of free thyroxine (fT4) and thyroid-stimulation hormone (TSH) did not correlate with those parameters. In terms of lipid metabolism, hypothyroidism is one of the causes of secondary obesity.

Nobiletin is shown to activate the nuclear receptors RORα and RORγ [44]. Both the receptors are highly expressed in skeletal muscles [78,79] and adipose tissues [80], suggesting their physiological roles in skeletal muscles and adipose tissues. RORα-deficient mice and *staggerer* mice (a natural mutant with defective RORα) displayed shorter hanging time [81], indicating impaired muscular strength. RORα was also shown to induce genes involved in fatty acid catabolism in C2C12 myoblastic cells [78]. In 3T3-L1 preadipocytes, expression of RORα was induced during adipogenesis, while adipocytic differentiation was suppressed by overexpressing RORα [80]. MEFs derived from *staggerer* mice displayed more pronounced lipid accumulation compared with MEFs derived from wild type littermates [80]. However, *staggerer* mice have less body weight than wild-type mice [82], instead they display severe atherosclerosis with decreased HDL level [82]. Abnormal production of inflammatory cytokines such as IL-1 and TNFα by peripheral macrophages of *staggerer* mice was shown [83], indicating RORα signaling may play suppressive role in atherosclerosis and sarcopenia mediated by chronic inflammation.

As described above, elamipretide was shown to have respiratory chain supercomplex assembly- promoting effect in the mitochondria derived from human heart muscle. There are some animal studies about its effect on skeletal muscle. In one report, treatment of aged female mice with elamipretide increased exercise tolerance without increase in mitochondrial content in skeletal muscle [84]. Another study reported increase of mitochondria function in skeletal muscle of heart failure-induced dogs [85].

## 5. Regulation of Mitochondrial Respiratory Chain Supercomplex Assembly by Exercise

It was shown that four months of exercise intervention led to increased mitochondrial respiratory chain supercomplex formation in healthy older men and women [86]. The formation of all five individual respiratory complexes was increased by exercise. In addition, it was observed that exercise redistributed these complexes in favor of I/III_2_/IV_n_-type supercomplex (respirasome). The results of this study suggested the existence of certain adaptive mechanisms involving the regulation of mitochondrial respiratory chain supercomplex assembly.

**Figure 2:**
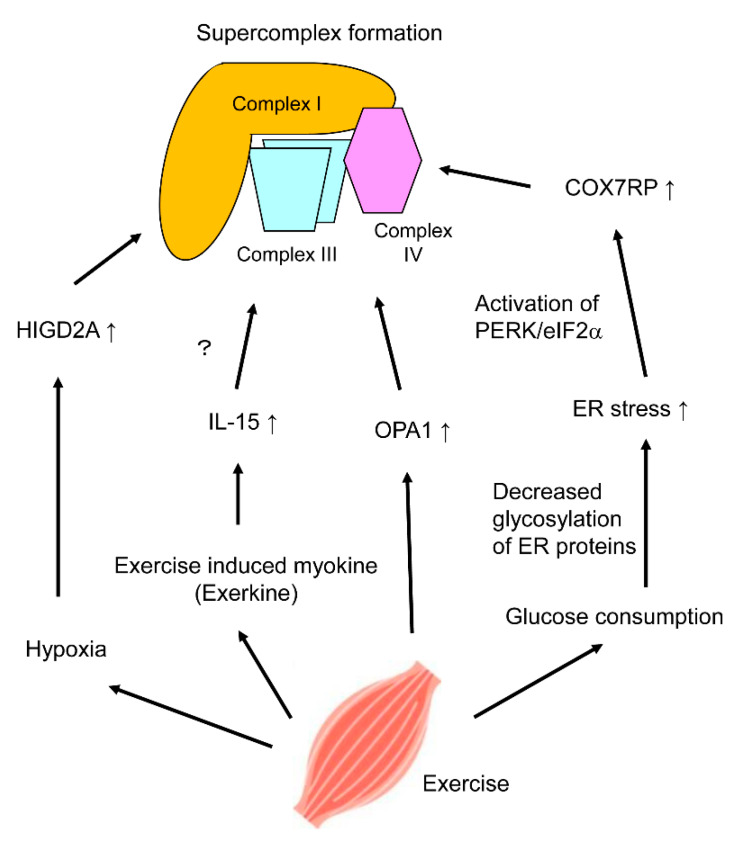
Putative mechanisms of mitochondrial respiratory chain supercomplex formation by exercise. Exercise intervention was shown to induce respiratory chain supercomplex formation [86]. Four putative regulatory mechanisms of respiratory chain supercomplex formation in skeletal muscle by exercise are shown. Exercise increases oxygen and glucose consumption, and induces secretion of myokines called ‘exerkines’. Hypoxia induces expression of HIGD2A (HIG1 hypoxia inducible domain family, member 2A), while insufficient glucose induces expression of COX7RP (cytochrome c oxidase subunit 7a-related polypeptide) through endoplasmic reticulum (ER) stress. Interleukin-15 (IL-15) is an exerkine which promotes mitochondrial respiratory chain supercomplex with an unknown mechanism. Routine exercise is shown to increase expression of OPA1 (optic atrophy 1) in skeletal muscles.

One of the mechanisms could be induction of HIGD2A by hypoxia [21], which can be induced by exercise (Figure 2). It is also noted that hypoxia decreased the formation of complex I-containing respiratory chain supercomplex in plant (potato) mitochondria [87]. However, the effect of hypoxia on mitochondrial respiratory chain supercomplex formation may depend on the expression of several supercomplex assembly factors; for example, overexpression of COX7RP in human breast cancer cells leads to the stabilization of complex I-containing supercomplex even in hypoxic conditions [23].

Another explanation for the adaptive regulation of mitochondrial respiratory chain supercomplex assembly could be attributed to the function of exerkines, which are exercise-induced myokines. The exerkine Interleukin-15 (IL-15) was shown to induce mitochondrial respiratory chain supercomplex formation [88]. In this study, acute treatment with supra-physiological concentration of IL-15 promoted complex III-containing supercomplex formation, while chronic treatment with physiological concentration of IL-15 promoted complex IV-containing supercomplex formation in myotubes derived from rat L6 myoblastic cells (Figure 2). The former treatment led to activation of AMPK (AMP-activated protein kinase), suggesting the underlying mechanism; however, no AMPK activation was observed in the latter condition. Moreover, chronic treatment with supraphysiological concentration of IL-15 impaired the formation of mitochondrial respiratory chain supercomplexes.

OPA1 which is important to maintain the shape of mitochondrial cristae contributes to respiratory chain supercomplex formation [34] as mentioned above. By comparison of elderly people with a sedentary lifestyle and senior sportsmen who routinely practice lifelong sports activities more than three times a week, significantly higher expression of OPA1 protein was observed in the skeletal muscles of senior sportsmen [52]. This study provides another putative link between exercise and increased mitochondrial respiratory chain supercomplex formation (Figure 2).

Recently, it was shown that low glucose condition triggers mitochondrial respiratory chain supercomplex formation. When U2OS cells, derived from human osteosarcoma, were cultured under glucose-deprived conditions (i.e., in galactose), notable increase in I/III_2_/IV_n_-type supercomplex (respirasome) assembly was observed [89]. AMPK inhibitor had no effect on mitochondrial respiratory chain supercomplex formation, indicating that galactose-induced respiratory chain supercomplex formation did not involve AMPK activation. Instead, glucose-deprived conditions seemed to increase endoplasmic reticulum (ER) stress due to the reduced amount of glycosylated proteins in ER. Inhibitor of PERK (PKR-like endoplasmic reticulum kinase) and eIF2α, the downstream target of PERK, attenuated mitochondrial respiratory chain supercomplex formation; while tunicamycin, an activator of ER stress, increased supercomplex formation. Moreover, ER stress induces the expression of *Cox7rp* [89], confirming the link between glucose deprivation and mitochondrial respiratory chain supercomplex formation (Figure 2). Although this study was not performed in the context of metabolism in skeletal muscle, the results could be applied for explaining the mechanism of adaptive promotion of mitochondrial respiratory chain supercomplex assembly caused by insufficient glucose supply during exercise.

## 6. Mitochondrial Respiratory Chain Supercomplex Assembly, Sarcopenia, and Aging

Sarcopenia is an age-dependent disorder of skeletal muscle. Decreased mitochondrial respiratory chain supercomplex formation in aged animals [51] implies that it can apply also in humans, and some age-dependent alterations in supercomplex assembly factors. In addition, molecules affecting mitochondrial respiratory chain supercomplex formation could be the cause.

One of the causes of aging is considered to be the accumulation of cellular and molecular damage over a lifetime. This damage is promoted mainly by ROS which are generated by metabolic and respiratory pathways. It is reported that ROS suppress formation of mitochondrial respiratory chain supercomplex [90]. It is also reported that ROS generation leads to loss of mitochondrial cardiolipin content [91], and cardiolipin content of mitochondria decreases in multiple tissues in many species [92]. Putting all these together, we can assume one of the causes of sarcopenia is impaired formation of mitochondria respiratory chain supercomplex mediated by loss of cardiolipin affected by ROS. This may explain the mechanism of the bioactive small molecules mentioned above, namely melatonin and coenzyme Q10, which function as radical scavenger and alleviate attacks of ROS toward cardiolipin (Figure 1).

As for destructive effect of ROS on mitochondrial respiratory chain supercomplex formation, another mechanism involving micro RNA is also proposed. miR-663 is a micro RNA which transduces signal from mitochondria to nuclei, and promotes mitochondria respiratory supercomplex formation as a result of its transcriptional regulation [93]. ROS are shown to suppress expression of miR-663, thus eventually suppress mitochondrial respiratory chain supercomplex formation.

In elderly people with sedentary lifestyle, age-associated decline in *OPA1* expression and its correlation with muscle weakness was observed as mentioned above [52]. In addition, in the process of human aging, three intrinsic bioactive small molecules introduced above are known to decrease, namely, coenzyme Q10 [94,95], T3 type of thyroid hormone [77], and melatonin [96]. This is also a candidate link between aging and decreased mitochondrial respiratory chain supercomplex formation.

## 7. Conclusions

In the present review, we described multiple factors involved in mitochondrial respiratory chain supercomplex formation (Figure 1), their roles in skeletal muscle and adipose tissues, potential promotive mechanism on respiratory chain supercomplex formation in skeletal muscle during exercise (Figure 2), and destructive mechanisms with aging. Since muscular tissues require much energy for their function, the mitochondrial respiratory chain supercomplex, which theoretically affects muscle quality, is one of the clinical targets for prevention or treatment of muscle disorders including sarcopenia. Promotive or destructive, clarifying the regulatory mechanisms will help to find therapeutic targets for conditions where mitochondrial respiratory chain supercomplex formation is related. Research direction could be discoveries of new molecules or pathways affecting known supercomplex assembly factors or molecules affecting supercomplex formation explained in this review, or discoveries of completely new assembly factors or affecting molecules. Small bioactive molecules mentioned in this review are attractive candidates for the future clinical use. However, most of the evidence of intrinsic substances is epidemiological, and there are no clinical studies on two exogenous compounds at present. Future intervention study targeting sarcopenia patients would be required to confirm the true relationship between mitochondrial respiratory chain supercomplex formation and muscle quality.
